# Betriebliches SARS-CoV-2-Risikomanagement zur wirkungsvollen Eindämmung von Infektionsketten: Methodik und Werkzeuge zur Frühintervention und Vorwärtsanalyse

**DOI:** 10.1007/s40664-022-00467-9

**Published:** 2022-06-09

**Authors:** Andreas Paaßen, Laura Anderle, Karsten John, Sebastian Wilbrand

**Affiliations:** 1 Gesundheitsmanagement Arbeitsmedizin Chemiepark Marl, Evonik Industries AG, Marl, Deutschland; 2 Mathematik in der Informatik, Westfälische Hochschule Gelsenkirchen Bocholt Recklinghausen, Gelsenkirchen, Deutschland

**Keywords:** Corona, Quarantänemanagement, Infektionskette, Risikobewertung, Epidemiologie, Teststrategie, Corona, Quarantine management, Infection chain, Risk assessment, Epidemiology, Test strategy

## Abstract

**Hintergrund:**

Während der SARS-CoV-2-Pandemie ist es vorrangig, die Mitarbeiter vor Infektionsrisiken zu schützen und die Geschäftstätigkeit zu sichern. Neue Virusvarianten mit erhöhter Ansteckungsgefahr erfordern eine weiterentwickelte Risikostrategie.

**Material und Methoden:**

Mehrere Standardmaßnahmen wie Tests, Isolierung und Quarantäne werden zu einer neuartigen Risikostrategie kombiniert. Epidemiologische Modellrechnungen und wissenschaftliche Erkenntnisse über den Verlauf der SARS-CoV-2-Infektiosität werden zur Optimierung dieser Strategie herangezogen. Das Verfahren ist in einem einfach zu bedienenden Rechner auf Excel-Basis implementiert.

**Aufbau in der Praxis und Ergebnisse:**

Alternative Maßnahmenkombinationen und praktische Aspekte werden erörtert. Anhand von Beispielrechnungen wird die Wirkung der diskutierten Maßnahmen demonstriert.

**Schlussfolgerung:**

Der aus diesen Grundlagen abgeleitete Quarantäne-Rechner ermöglicht es auch Nicht-Fachleuten, eine differenzierte Risikoanalyse durchzuführen und optimierte Maßnahmen einzuleiten. Gezielte Prüfroutinen und alternative Maßnahmen sichern die Personalverfügbarkeit.

## Abstract (en)

**Background:**

During the severe acute respiratory syndrome coronavirus 2 (SARS-CoV-2) pandemic it is a priority that employees need to be protected from infection risks and business activities need to be ensured. New virus variants with increased infection risks require an advanced risk strategy.

**Material and methods:**

Several standard measures, such as testing, isolation and quarantine are combined to form a novel risk strategy. Epidemiological model calculations and scientific knowledge about the course of SARS-CoV‑2 infectivity are used to optimize this strategy. The procedure is implemented in an easy to use calculator based on Excel.

**Layout in practice and results:**

Alternative combinations of measures and practical aspects are discussed. Examples of calculations are used to demonstrate the effect of the discussed measures.

**Conclusion:**

The quarantine calculator derived from these principles enables even nonspecialists to perform a differentiated risk analysis and to introduce optimized measures. Targeted testing routines and alternative measures ensure staff availability.

In den Betrieben haben während der Pandemie der Schutz der Beschäftigten und Betriebsfremden sowie die Sicherstellung der Geschäftsaktivität Priorität. Hohe Neuinfektionszahlen können die personellen Ressourcen von Gesundheitsämtern zur Kontaktnachverfolgung überfordern [1, 2]. Die dadurch entstehenden Zeitverzögerungen in der Kontaktnachverfolgung bergen insbesondere an nicht Homeoffice-fähigen Arbeitsplätzen das Risiko innerbetrieblicher Infektionsketten [3]. Die dabei in kurzer Zeit verursachte hohe Anzahl an krankheitsbedingten Ausfällen und Isolationsmaßnahmen stellen eine große Bedrohung für die Weiterführung der Geschäftsaktivität dar [4]. Die neuen Virusvarianten erfordern mit ihrer Fähigkeit, eruptive Ausbrüche zu verursachen, eine weiterentwickelte Risikostrategie.

Zwei wichtige Säulen bei der Bewältigung sind die Minimierung der Übertragungsrisiken durch wirksame Schutzmaßnahmen und ein möglichst effizientes Isolations- und Quarantänemanagement. Dabei verfügen Betriebe über sehr effektive Interventionsmöglichkeiten wie die verbindliche Anordnung von Schutzmaßnahmen sowie auch den möglichen Verzicht auf die Arbeitsleistung vor Ort und damit die Herausnahme von innerbetrieblichen Risiken. Wie in dieser Arbeit gezeigt wird, gelingt es mit diesen Interventionsmöglichkeiten und einem erweiterten Risikomanagement trotz der mit Tätigkeiten verbundenen Sozialkontakte, die Ansteckungsrisiken für Beschäftigte im Vergleich mit außerbetrieblichen Risiken sehr gering zu halten.

Das hier vorgestellte erweiterte Isolations- und Quarantänemanagement unter besonderer Berücksichtigung der neuen Virusmutationen basiert auf einer epidemiologischen Modellierung, um Risiken genau einschätzen zu können. Es wurde im praktischen Einsatz bei mehr als 200 Fällen während der Alpha-Welle (B 1.1.7) 2020/2021 im Chemiepark Marl, einem großen deutschen Chemiestandort mit insgesamt ca. 10.000 Beschäftigten und ca. 3000 Beschäftigten von Partnerfirmen bei großen Investitionsprojekten, entwickelt. Das Isolations- und Quarantänemanagement im Chemiepark Marl wird zentral vom Werksärztlichen Dienst der Evonik Industries AG für eine Vielzahl von Gesellschaften, Firmen und Subunternehmen gesteuert, überwacht und mit den zuständigen Behörden abgestimmt. Dazu wurde ein digitalisiertes Prozessmanagement als sog. „Corona Control Center“ eingerichtet.

Um die Wirkung der vorgestellten Methodik nachvollziehbar darzustellen, haben wir uns mit den Beispielberechnungen auf die klassisch verlaufende Alpha-Varianten-Welle bezogen. Bei den zu einem späteren Zeitpunkt durchgeführten Impfungen steigt die Komplexität der Berechnung durch die Berücksichtigung der aktuellen Impfeffektivität an und eignet sich deshalb weniger zur Darstellung der Methodik. In der Praxis haben wir das Rechentool jeweils an die aktuellen Virusvarianten und anderen Rahmenbedingungen angepasst.

Betriebe sind beim Quarantänemanagement einem Zielkonflikt ausgesetzt: Werden für eine möglichst effektive Isolationsmaßnahme zu viele Mitarbeiter auf Initiative des Betriebs freigestellt, so sind Produktion und Geschäftsaktivitäten unmittelbar gefährdet. Wird auf ein über staatliche Vorgaben hinausgehendes Quarantänemanagement verzichtet, erhöht sich das Risiko für innerbetriebliche Infektionsketten mit großen Personalausfällen. Um zwischen diesen beiden Extremfällen abzuwägen und ein dem Einzelfall angepasstes Vorgehen zu erreichen, ist das Isolations- und Quarantänemanagement, insbesondere bei besorgniserregenden Virusvarianten, um erweiterte Maßnahmen und Alternativmaßnahmen zu ergänzen.

Aufgrund des Verlaufs der Infektiosität von Erkrankten (vgl. Abschnitt „Methodik“) ist eine sehr kurze Reaktionszeit bis zur Einleitung von Maßnahmen ein entscheidender Erfolgsfaktor. Diese darf jedoch nicht auf Kosten der Zielgenauigkeit der Maßnahmen gehen. Deshalb wurde zum arbeitsplatznahen Einsatz ein auf einer epidemiologischen Modellierung basierendes Rechentool entwickelt, das bei der routinierten und evidenzbasierten Ermittlung geeigneter Maßnahmen unterstützen kann.

Die Maßnahmen Isolation als Absonderung von Infektiösen und die Quarantäne als Absonderung von Kontaktpersonen werden im Weiteren nicht unter rechtlichen Aspekten betrachtet. Diese Maßnahmen können für den Betroffenen verpflichtend durch eine Allgemeinverfügung des Landes oder durch behördliche Anordnung sein. Der Arbeitgeber kann seinen Beschäftigten von der Arbeitsleistung freistellen oder mobile Arbeit zuweisen. Im häuslichen Umfeld kann freiwillig eine Selbstquarantäne eingehalten werden. Behördliche Anordnungen gelten in jedem Fall vorrangig, können und sollen aber durch freiwillige und durch arbeitgeberveranlasste Maßnahmen ergänzt werden.

## Methodik

Die mathematischen Berechnungen des Isolations- und Quarantänemanagements basieren auf der Grundlagenarbeit von Lipsitch et al., 2003, Department of Epidemiology, Harvard School of Public Health [5] und der epidemiologischen Modellierung von Ferretti et al., 2020, Big Data Institute, University of Oxford [6].

Der Reproduktionsfaktor *R* beschreibt, wie viele Menschen eine infizierte Person im Mittel ansteckt. Er berechnet sich nach Lipsitch et al. [5] als:

1 $$R_{0}=k\cdot b\cdot D$$

aus der Anzahl von Einzelkontakten *k*, der Übertragungswahrscheinlichkeit *b* der Kontakte sowie der mittleren Dauer der Infektiosität *D*.

Der Erfolg einer Isolations- und Quarantänemaßnahme ist definiert als [5]:

2 $$R_{\mathrm{int}}=D_{\mathrm{int}}\cdot k_{\mathrm{int}}\cdot b_{\mathrm{int}}=R_{0}\cdot \frac{D_{\mathrm{int}}}{D}\cdot \left(1-\mathrm{q}\right)$$

Der Erfolg (*R*_int_) einer Isolations- und Quarantänemaßnahme hängt somit von der Ansteckungsfähigkeit des Virus (Basisreproduktionszahl *R*_0_), der durch die Intervention erfolgten Reduktion von infektiösen Tagen mit Kontakten (*D*_*int*_: verbliebene infektiöse Tage nach Intervention) und dem prozentualen Anteil der verbleibenden Infektionswahrscheinlichkeit pro Zeiteinheit an der ursprünglichen Infektionswahrscheinlichkeit q bei $1-\mathrm{q}=\frac{k_{\mathrm{int}}\cdot b_{\mathrm{int}}}{k\cdot b}$ ab.

Diese Formeln gelten für Erkrankungen, bei denen die Infektiosität während des Verlaufes konstant ist. Bei SARS-CoV‑2 liegen jedoch im Verlauf erheblich tagesdifferente Ansteckungswahrscheinlichkeiten vor. Aus der epidemiologischen Modellierung von Ferretti et al. [6] kann die Ansteckungswahrscheinlichkeit an den einzelnen Tagen berechnet werden. Dabei kann die Generationszeit (also die Dauer von Infektion zu Folgeinfektion in Tagen) bei COVID-19 als Weibull-verteilt mit Form-Parameter $k=2,886$ und Skalenparameter $\frac{1}{\lambda }=5,665$ angenommen werden. Daraus ergibt sich für die durchschnittliche Infektiosität die folgende Dichtefunktion (*τ*) (mit $\tau > 0$, wobei $\tau =0$ dem Zeitpunkt der Infektion entspricht):

3 $$\beta \left(\tau \right)=\lambda k\left(\lambda \tau \right)^{k-1}e^{-{\left(\lambda \tau \right)^{k}}},$$

was die kumulierte Infektionswahrscheinlichkeit

4 $$B_{\left[t_{1},t_{2}\right]}={\int }_{t_{1}}^{t_{2}}\beta \left(\tau \right)d\tau$$

im Zeitintervall $\left[t_{1},t_{2}\right]$ (in Tagen seit dem Infektionszeitpunkt der Quelle) ergibt. Die Ansteckungswahrscheinlichkeiten werden ab Infektionszeitpunkt berechnet, der Symptombeginn wird am 5. Tag angenommen.

In der Praxis ist der konkrete Infektionszeitpunkt jedoch nicht bekannt. Beobachtbar ist lediglich der Beginn von Krankheitssymptomen bei einem klassischen Verlauf, ersatzweise ein positives Testergebnis. Deshalb ist es sinnvoll, den Krankheitsverlauf vom Zeitpunkt des beobachtbaren Symptombeginns zu bestimmen und den Infektionszeitpunkt über die mittlere Inkubationszeit theoretisch zu berechnen. Als Grundlage für die Kalkulation des Krankheitsverlaufes und der tagesdifferenten Infektionsrisiken wird daher der Symptombeginn verwendet, der im Folgenden als Tag 0 gesetzt wird. Wenn diese Information nicht vorliegt, wird ersatzweise der Termin der Testung am Tag 0 oder die Mitteilung des positiven Testergebnisses an Tag 1 gesetzt. Dies betrifft insbesondere Testbefunde bei prä- oder asymptomatischen Fällen.

**Figure 1:**
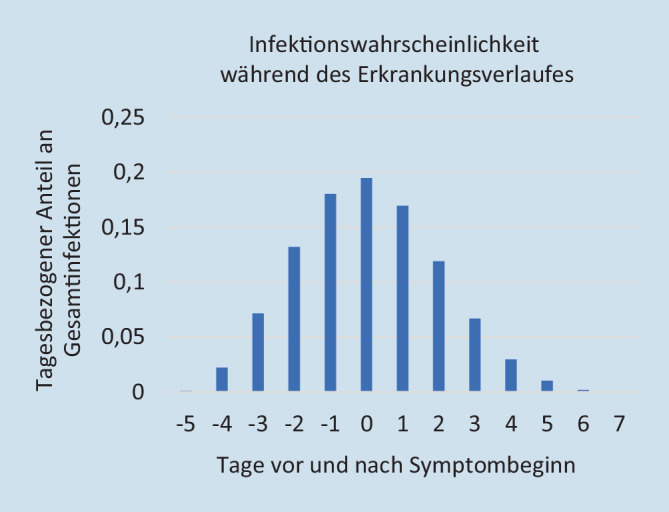

Im Arbeitsumfeld sind anders als in einem Laborumfeld Ungenauigkeiten bei dem Zeitpunkt der oft protrahierten Symptomentwicklung oder ihrer Wahrnehmung zu konstatieren. Um unter diesen Rahmenbedingungen eine praxisnahe Anwendung des Rechentools zu erreichen, werden die Ereignisse Infektionszeitpunkt und Symptombeginn rechnerisch auf den Mittag normiert und die Infektionswahrscheinlichkeiten auf Wochen- bzw. Arbeitstage kumuliert (Abb. 1).

Da der Symptombeginn nach Ferretti et al. [6] am 5. Tag angenommen wird, muss Gl. 4 dementsprechend angepasst werden (mittels der Variablensubstitution *T* = *t* − 5 zum Zeitpunkt *T* ab Symptombeginn):

5 $$B_{\left[T_{1},T_{2}\right]}={\int }_{T_{1}+5}^{T_{2}+5}\beta \left(\tau \right)d\tau$$

Hierbei stellt sich die Frage, ob diese am Symptombeginn orientierte Verteilung durch die Länge der Inkubationszeit beeinflusst wird. He et al. [7] konnten zeigen, dass die Wahrscheinlichkeit unabhängig von der Inkubationszeit bis zum Tag −4 sehr niedrig bleibt und erst am Tag −3 einen steilen Anstieg erfährt. Somit ist die Berechnung der Wahrscheinlichkeiten unabhängig von der individuellen Inkubationszeit in sehr guter Näherung gültig.

Die Effektivität *E*_*int*_ der jeweiligen Isolations- oder Quarantänemaßnahme errechnet sich aus der Anpassung der Gln. 2 und 4 an tagesdifferente Ansteckungsrisiken zu:

6 $$E_{\mathrm{int}}=\frac{B_{\left[i,7\right]}}{B_{\left[-5,7\right]}}$$

i: Tag nach Durchführung der Absonderungsmaßnahme

Die Reproduktionszahl nach den jeweiligen Stufen von Isolation und Quarantäne ergibt sich aus der Anpassung der Gl. 2 zu:

7 $$R_{\mathrm{int}}=R_{0}{\sum }_{d=i}^{7}B_{d}\cdot ({\sum }_{k=1}^{K_{d}}P_{k})$$

*R*_*int*_: Reproduktionszahl nach Intervention
*R*_0_: Basisreproduktionszahl
*K*_*d*_: Kontakte am Tag d
*P*_*k*_: Übertragungswahrscheinlichkeiten des jeweiligen Kontaktes
$$B_{d}=B_{[d-0,5;d+0,5]}$$:
kumulierte Infektiosität am Tag d

Gl. 7 ist eine Verfeinerung von Gl. 2, da die tagesspezifische Infektiosität berücksichtigt wird (${\sum }_{d=i}^{7}B_{d}< 1$ ist daher der deutlich präzisere Ersatz für $\frac{D_{\mathrm{int}}}{D}$). Statt wie in Gl. 2 die gesamte Reduktion der Ansteckungswahrscheinlichkeit $(1-\mathrm{q})$, wird in Gl. 7 mit der durch die getroffenen Maßnahmen verringerten Anzahl *K*_*d*_ an tagesspezifischen Kontakten sowie deren kontaktspezifischer Ansteckungswahrscheinlichkeit gerechnet.

Die Ermittlung von sozialen Kontakten wird durch Schichtpläne bzw. Tätigkeitsdokumentationen und durch die Befragung der Beschäftigten durchgeführt. Die Beschäftigten führen teilweise auf freiwilliger Basis Kontakttagebücher, die sie als persönliche Gedächtnisstütze nutzen. In Einzelfällen kommen auch digitale Medien wie Trackersysteme als ergänzende Informationsquelle in Frage. Soziale Kontakte werden als Aufenthalt innerhalb des sozialen Mindestabstandes oder als gemeinsamer Aufenthalt in Innenräumen definiert. Am Arbeitsplatz sind die sozialen Kontakte vielfältig und häufig länger andauernd. Unter Berücksichtigung der Hauptfaktoren Kontaktdistanz, Dauer, Aktivität, Schutzmaßnahmen und gemeinsamer Innenraumnutzung wurden die Übertragungsrisiken nach allgemeinen Empfehlungen der Gesundheitsbehörden [8] und Studien zur Transmissibilität, Wirksamkeit von Schutzmaßnahmen und Aerosolen in Innenräumen [9–12] für betriebsspezifische Transmissionsbedingungen eingeschätzt. Die Übertragungsrisiken wurden 3 Gruppen mit 0,2 für hohe, 0,1 für mittlere und 0,05 für geringe Risiken zugeordnet. Diese Werte sind für andere Transmissionsbedingungen gegebenenfalls anzupassen.

Die Höhe der Basisreproduktionszahl *R*_0_ weist in verschiedenen Studien eine hohe Unsicherheit auf [13–16]. Für die Berechnungen wird ein *R*_0_ beim Wildtyp von 2,5 verwendet, was der bestverfügbaren Schätzung der CDC (Centers for Disease Control and Prevention, Behörde des US-amerikanischen Gesundheitsministeriums) in ihren aktuellen Pandemie-Planung-Szenarios entspricht [17]. Bei Virusvarianten wird *R*_0_ mit der aktuell geschätzten Infektiosität multipliziert [18–20] (B 1.1.7 $R_{0}=3,75$). Die zu erwartenden Rückgänge der Basisreproduktionszahl durch die zunehmende Immunisierung der Bevölkerung ist in der Zukunft durch Anpassung der *R*_0_ zu berücksichtigen.

Für Testungen wurde der SARS-CoV‑2 Rapid Antigen Test von SD Biosensor, Korea, vertrieben durch Roche Diagnostics GmbH mit einer vom Hersteller angegebenen Sensitivität von 96,52 % (95 % KI 91,33–99,04 %) und einer Spezifität von 99,68 % (95 % KI 98,22–99,99 %) verwendet. Die Proben wurden mit tiefen nasopharyngealen Abstrichen gewonnen.

## Ergebnisse und Ausgestaltung

Beim Vergleich einzelner Strategien zur Eindämmung von Ausbrüchen wie Isolation, Kontaktverfolgung mit Quarantäne, Symptommonitoring und Teststrategien zeigt sich in der Literatur, dass Kombinationen verschiedener Maßnahmen erheblich effektiver sein können als die Einzelmaßnahmen [21, 22]. Um die erhöhte Komplexität eines solchen erfolgsoptimierten Vorgehens beherrschen zu können, wurde auf Grundlage der epidemiologischen Modellierung ein ChainCUT-Quarantänerechner auf Excel-Basis entwickelt. Nach Erfassung der ermittelten Kontakte wird die Effektivität der Interventionen berechnet und Ansteckungsrisiken für die spezifische betriebliche Situation und Virusvariante analysiert. Aus dieser Risikoanalyse abgeleitet werden kombinierte Maßnahmen zur Eindämmung innerbetrieblicher Infektionen berechnet.

**Figure 2:**
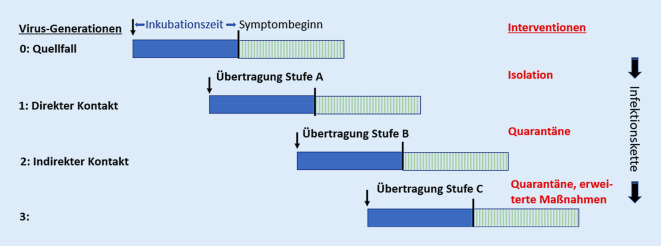

Risikobetrachtungen und erweiterte Maßnahmen werden deshalb nicht nur für den Quellfall als Virusgeneration 0 und die direkten Kontaktpersonen als Generation 1, sondern auch für die indirekten Kontaktpersonen (Generation 2) durchgeführt. Das sind die Personen, die keinen Kontakt mit dem Quellfall, sondern Kontakt mit direkten Kontaktpersonen hatten. Durch die Ausweitung der Betrachtung und Interventionen kann eine weitere Ausbreitung (Generation 3) auch bei Virusvarianten mit erhöhter Infektiosität verhindert werden (Abb. 2).

Um möglichst alle Risiken durch Kontakte zu erfassen, wird die Kontakterfassung auf den Tag −3, einem Tag mit mittelhoher Infektiosität, ausgeweitet (Abb. 1).

Bei gemeinsamen Aufenthalten im Arbeits- oder Sozialbereich sind Ansteckungsrisiken auch bei Einhaltung des Mindestabstandes nicht auszuschließen. Unter Sozialbereich werden gleichzeitige Aufenthalte in Gemeinschaftsräumen wie Pausen‑, Raucher‑, Wasch- oder Umkleideräumen verstanden. Hierbei werden geringe Risiken durch den Aufenthalt in gleichen Innenräumen, gelegentliche kurzzeitige Begegnungen, akzidentelle Expositionen durch Luftströmungen oder auch Schmierinfektionen angenommen und überschlägig berücksichtigt.

Der ChainCUT-Quarantänerechner visualisiert im Risikoanalyse-Dashboard die Effektivität der Absonderungsmaßnahme nach Gl. 6 und die Reproduktionszahlen nach Intervention nach Gl. 7 (Abb. 3, 4, 5 und 6) getrennt nach Interventionsstufen. Damit lassen sich Maßnahmen zielgenau steuern.

Als bestimmender Erfolgsfaktor für ein Isolations- und Quarantänemanagement konnte durch Beispielrechnungen der Zeitverzug bei der Einleitung von Maßnahmen identifiziert werden. Eine Beispielrechnung geht von täglich 4 direkten Kontakten des Quellfalls mit einem Gesamtübertragungsrisiko von 0,6 (2 mit hohem, 2 mit mittlerem Übertragungsrisiko) und jeweils 2 indirekten Kontakten mit einem Übertragungsrisiko von 0,3 aus (Abb. 3, 4, 5 und 6). Diese Rechnung wird für die Zeitpunkte Einleitung von Interventionsmaßnahmen am Tag 0, am Tag 3 und am Tag 5 durchgeführt. Tag 0 ist der Tag, an dem erste Symptome auftreten. Der Zeitverzug bis Tag 3 ist der typische Fall, wenn das Ergebnis des PCR-Tests abgewartet wird und dann eine Intervention erfolgt. Wenn diese Intervention zusätzlich verzögert stattfindet, ist häufig Tag 5 erreicht.

Für die Beispielrechnung wird die im Abschnitt „Methodik“ abgeleitete Basisreproduktionszahl von 3,75 für die Virusvariante B 1.1.7 (Alpha) verwendet. Sie zeigt für die Frühintervention am Tag 0 trotz der umfangreichen Kontakte einen Reproduktionsfaktor für den Quellfall (Stufe A) nach Isolationsmaßnahme von 2,25, für den direkten Kontakt (Stufe B) nach Kontaktquarantäne von 1,30 und für den indirekten Kontakt (Stufe C) nach Kontaktquarantäne von 0,31 (App. Abb. 3). Damit liegt der R‑Faktor in Stufe C deutlich unter 1, und das Risiko des Auftretens einer Infektionskette ist sehr gering. Schon bei einem Zeitverzug mit einem Interventionsbeginn am Tag 3 beträgt der R‑Faktor bei fortgesetzten Kontakten für die Stufe C 1,78 und zeigt somit die Gefahr einer fortgesetzten Infektionskette an (App. Abb. 4). Am Tag 5 beträgt der R‑Faktor für Stufe C schon 2,31 (App. Abb. 5). Damit ist eine Eingrenzung der Gefährdung auf einen kleinen Personenkreis kaum noch möglich.

Auch bei Selbstisolierung des Quellfalls am Tag 0 und ohne weitere Kontakte mit den direkten Kontaktpersonen wirkt sich der Zeitverzug durch die fortbestehenden Kontakte der direkten mit den indirekten Personen auf den R‑Wert der Stufe C am Tag 3 von 1,10 und am Tag 5 von 1,37 aus (App. Abb. 6). Dies bedeutet, dass jeder Zeitverzug bei der Einleitung von Maßnahmen die Chancen einer frühzeitigen Eindämmung eines Infektionsgeschehens deutlich verschlechtert, das gilt in besonderem Maße für Virusvarianten mit erhöhter Infektiosität.

Die Aussagefähigkeit der berechneten Reproduktionswerte lässt sich durch eine Kombination mit einer differenzierten gezielten Teststrategie als erweiterte Maßnahme erheblich verbessern (App. Abb. 3, 4 und 5*gepunktete Linie*). Dabei wird bei direkten Kontaktpersonen am Tag der Einleitung der Maßnahmen die Durchführung einer Corona-Testung angestrebt. Dafür kommt grundsätzlich entweder ein PCR-Test oder ein Antigen-Schnelltest in Frage. PCR-Tests stellen den Goldstandard dar, da sie eine hohe Sensitivität aufweisen. Dabei treten in der Praxis jedoch je nach Auslastung der Laborkapazitäten Verzögerungen von 2 bis 3 Tagen bis zum Vorliegen der Ergebnisse auf. Damit kann das Ziel, Infektionsketten durch schnelle Maßnahmeneinleitung zu vermeiden, nicht erreicht werden. Deshalb wird ein Antigen-Schnelltest verwendet, bei dem jedoch eine geringere Sensitivität zu erwarten ist. Die Qualitätskriterien [23], die die Sensitivität eines Antigen-Schnelltestes wesentlich mitbestimmen, werden durch die nasopharyngeale Probennahme durch geschultes Personal und eine sorgfältige Durchführung gewährleistet. Die damit zu erwartende Genauigkeit des Antigen-Schnelltestes ist für die Fragestellung einer aktuell vorliegenden Infektiosität der direkten Kontaktperson ausreichend [23, 24]. Dies betrifft insbesondere die Sensitivität bei hoher Infektiosität mit Ct-Werten < 30 [25].

Damit kann mit ausreichend hoher Sicherheit festgestellt werden, ob die direkten Kontaktpersonen zu Beginn ihrer Quarantäne infektiös sind. Im Fall von negativen Testergebnissen kann eine Übertragung auf die jeweiligen indirekten Kontaktpersonen in der Praxis nahezu ausgeschlossen werden. Sind alle Testergebnisse der direkten Kontakte negativ (R-Wert in Stufe C 0,00), kann mit hoher Sicherheit die Beendigung der Infektionskette festgestellt werden. In diesem Fall sind keine Quarantänemaßnahmen für die indirekten Kontakte einzuleiten. Das negative Testergebnis der direkten Kontaktpersonen schließt ihre Infektiosität zu diesem Zeitpunkt mit in der Praxis hinreichender Genauigkeit aus. Dabei hat die Schnelltestung ausdrücklich nicht das Ziel, eine Infektion auszuschließen und damit eine Quarantäne für die direkten Kontakte zu vermeiden, sondern lediglich eine Aussage über deren Infektiosität zu treffen und somit das behördliche Risikomanagement zu erweitern.

Bei unsicheren Angaben bei der Kontaktbefragung oder einer hohen Anzahl von Personen ohne direkten Kontakt im gleichen Arbeits- und Sozialbereich ist ein erhöhtes Risiko einer Ansteckung, insbesondere bei kritischen Virusvarianten, in dieser Gruppe möglich. Deshalb kann hier eine befristete regelmäßige Antigen-Schnelltestung der gesamten Gruppe erforderlich sein.

## Limitationen und Unsicherheiten

Die Berechnungen der Infektionswahrscheinlichkeiten orientieren sich am Symptombeginn. In der Praxis ist allerdings der Symptombeginn nicht immer bekannt. Ursache dafür kann die fehlende Übermittlung dieser Daten durch den Betroffenen oder symptomlose Testungen aus unterschiedlichem Anlass sein. Die Bestimmung von Tag 0 ist so mit Unsicherheiten verbunden. Eine Fehlbestimmung kann dazu führen, dass die Risiken an den hochinfektiösen Tagen (Tage −2 bis +1) nicht vollständig berücksichtigt werden können und damit die Effektivität der Maßnahmen abnimmt. Die systematische Berücksichtigung des Tag −3 kann diesen Effekt verkleinern.

Bei der Ermittlung von Kontakten durch Befragung sind die Motivation und das Erinnerungsvermögen der Beschäftigten von Bedeutung. Die Einschränkung des Erinnerungsvermögens kann jedoch durch sofortige Einleitung der Maßnahmen sowie durch Hinzuziehen von Schichtplänen und Tätigkeitsdokumentationen reduziert werden. Die Motivation, einen innerbetrieblichen Kontakt mitzuteilen, kann jedoch eingeschränkt sein, wenn beispielsweise angeordnete Schutzmaßnahmen vom Beschäftigten nicht eingehalten wurden und aus Sicht des Beschäftigten keine ausreichende betriebliche Vertrauenskultur besteht.

Die Einschätzung der Virusaufnahme eines Empfängers nach den Transmissionsbedingungen und Wirkung der Schutzmaßnahmen bestimmt das Übertragungsrisiko und kann insbesondere im Arbeitsumfeld sehr komplex sein. Zur Einschätzung werden in der Regel einfache Schemata der Gesundheitsbehörden verwendet, die im Wesentlichen den Abstand und die Dauer eines Kontaktes berücksichtigen. Vielfach zu beobachten ist, dass Distanzen in der Regel überschätzt, Kontaktdauern eher unterschätzt werden. Eine besondere Herausforderung stellt die kumulative Betrachtung verschiedener Kontakte, Transmissionsbedingungen und Innenraumbelastungen für die gesamte Arbeitsdauer dar. Insgesamt ist nach praktischer Erfahrung mit einer häufigen Fehleinschätzung der Übertragungsrisiken zu rechnen. Die Entwicklung und der Einsatz praxistauglicher Transmissionssimulatoren können hier zu erheblichen Verbesserungen führen.

## Diskussion

Die sichere Vermeidung von betrieblichen Infektionsketten, insbesondere bei Virusvarianten mit erhöhter Infektiosität, setzt voraus, dass Risiken vollständig ermittelt und bewertet werden. Die qualifizierte Risikoanalyse identifiziert, wie gezeigt, praxistaugliche und erfolgreich erprobte Maßnahmen, um Ausbrüche frühzeitig zu begrenzen und Maßnahmen durch Gesundheitsämter arbeitgeberseitig gezielt zu ergänzen, um den Kreis der Gefährdeten klein zu halten. Die vorgestellte epidemiologische Modellierung berechnet Ansteckungsrisiken und den voraussichtlichen Erfolg von Maßnahmen über Virusgenerationen hinweg. Die Aussagefähigkeit dieser Berechnungen lässt sich durch eine Kombination mit einer differenzierten gezielten Teststrategie deutlich steigern.

Die Erfassung von betrieblichen Kontakten mit der infektiösen Quelle ist die Grundlage zur Berechnung der kumulierten Übertragungsrisiken für jedes Quelle-Empfänger-Paar. Eine klassische Kontaktverfolgung wählt die Personen für eine Quarantäne nach dem Ereignis eines Hochrisikokontaktes, definiert durch die ungeschützte Dauer innerhalb des sozialen Mindestabstandes oder nach gemeinsamer Aufenthaltsdauer in einem Raum, aus [8]. Dabei werden Risiken durch Mehrfachkontakte an verschiedenen Tagen auch unterhalb der Definition des Hochrisikokontaktes systematisch nicht berücksichtigt. Dies führt zu Lücken in der Risikobewertung und nachfolgend in der Auswahl von Maßnahmen. Das vorgestellte Modell bewertet kumuliert alle Kontakte in Abhängigkeit von der Höhe des jeweiligen Übertragungsrisikos und der tagesabhängigen Infektiosität. Dabei werden auch Kontakte am Tag −3 mit mittelhoher Infektiosität berücksichtigt. Damit entsteht ein vollständiges Bild der Risiken für jedes Quelle-Empfänger-Paar.

Bei Infektionserkrankungen mit einer hohen Infektiosität vor Symptomeintritt beschreiben epidemiologische Arbeiten [26, 27] die große Bedeutung des Zeitverzuges bis zur Einleitung der Maßnahmen für die Kontrolle von Ausbrüchen. Die Ergebnisse der Beispielrechnung nach Abb. 3, 4, 5 und 6 bestätigen diesen Zusammenhang. Zum Zeitpunkt des Auftretens erster Symptome bei der Quelle am Tag 0 muss abhängig von der Anzahl der Kontakte und ihrer Übertragungswahrscheinlichkeit bereits mit der Übertragung von Virusgeneration 0 (Quelle) auf die Generation 1 (direkte Kontakte) gerechnet werden (Abb. 2). Eine weitere Übertragung auf die Generation 2 (indirekte Kontakte) ist zu diesem Zeitpunkt noch unwahrscheinlich. Die Interventionen zu diesem Zeitpunkt sind deshalb sehr erfolgversprechend und weisen in der Beispielrechnung für die indirekten Kontakte für B 1.1.7-Reproduktionswerte deutlich kleiner als 1 aus.

Jeder weitere Tag bis zur Einleitung der Interventionen erhöht die Wahrscheinlichkeit der Übertragung auf die Generation 2 (indirekte Kontakte). So zeigt die Beispielrechnung R‑Werte am Tag 3 von 1,78 bei fortgesetzten Kontakten und am Tag 5 schon 2,31 für B 1.1.7 an. Damit wird der Kreis der Infektionsgefährdeten und auch der Umfang erforderlicher Maßnahmen deutlich ausgeweitet.

Gesundheitsbehörden werden in der Regel über positive PCR-Testungen informiert und werden danach aktiv. Eine Verzögerung von 3 Tagen nach Symptombeginn muss in der Praxis schon als Idealfall betrachtet werden. Die eingeleiteten Interventionen beziehen sich in der Regel auf die Quelle und die direkten Kontaktpersonen. Da zu diesem Zeitpunkt schon mit einer Übertragung auf die nächste Generation gerechnet werden muss, kann mit einer sicheren Beendigung der Infektionskette nicht gerechnet werden. Dies gilt insbesondere für die Virusvarianten mit erhöhter Infektiosität.

Deshalb ist eine möglichst frühzeitige Einleitung von Maßnahmen durch den Betrieb, insbesondere bei den neuen Virusvarianten, ein entscheidender Erfolgsfaktor. Die Durchführung einer sofortigen Antigen-Schnelltestung im Verdachtsfall muss dabei betrieblich veranlasst werden. Das Abwarten von Testungen und Ergebnissen durch das Gesundheitssystem führt in der Praxis häufig zu erheblichen Zeitverzögerungen.

Der Einsatz von betrieblichen Trackingsystemen oder Kontaktprotokollen kann die Identifizierung der Kontakte unterstützen. Allerdings ist allein durch den Einsatz dieser Systeme noch kein effizientes Risikomanagement gegeben, da die Faktoren „Reaktionszeit bis zur Einleitung von Maßnahmen“ und „Berücksichtigung aller Risiken“ den Erfolg der Maßnahmen überwiegend bestimmen.

Erweiterte Maßnahmen als gezielte differenzierte Teststrategie der direkten Kontaktpersonen zu Beginn ihrer Quarantäne sind bei einem erhöhten Reproduktionswert der Stufe C erforderlich. Mit der Schnelltestung der direkten Kontaktpersonen kann bei negativen Ergebnissen eine Übertragung auf die jeweiligen indirekten Kontaktpersonen und eine Infektionskette mit hoher Sicherheit ausgeschlossen werden. Bei positivem Ergebnis können Quarantänemaßnahmen auf die betroffenen indirekten Kontakte eingegrenzt werden. Mit diesen erweiterten Maßnahmen lässt sich so der Kreis der gefährdeten Personen erheblich einschränken. So können Personalressourcen geschont und umfangreiche weitere Quarantänen oder Testungen vermieden werden.

Bei unübersichtlichen betrieblichen Kontaktverhältnissen mit vielen Personen im Arbeits- oder Sozialbereich oder unzuverlässigen Angaben kann die Identifikation von Risikopersonen nicht mit hinreichender Sicherheit erfolgen. Insbesondere bei den besorgniserregenden Virusvarianten kann neben den Quarantänemaßnahmen identifizierter Kontaktpersonen eine befristete Gruppentestung für diesen Bereich eingesetzt werden. Grundsätzlich sollen Testungen wegen der besseren Aussagefähigkeit und Vorhersagewerte gezielt für Personen und Gruppen mit erhöhter Ansteckungsgefährdung durchgeführt werden.

Wenn die betriebliche Personalverfügbarkeit durch Quarantänemaßnahmen kritisch abnimmt, können alternative Maßnahmen zur Quarantäne durchgeführt werden. Eine Alternative ist die Organisation von Einzel- oder Isolierarbeit. Dies erfordert eine räumliche und organisatorische Abgrenzung des Arbeitsplatzes ohne soziale Kontakte. Wenn dies nicht möglich ist, kann eine vorarbeitstägliche Schnelltestung als Ersatz für die Quarantäne durchgeführt werden. Dabei sind besondere Anforderungen an Qualität und Aussagefähigkeit der Schnelltestung sowie an eine fachgerecht durchgeführte Probenahme zu stellen. Wegen der dabei bestehenden Restrisiken ist die lückenlose Einhaltung der Basisschutzmaßnahmen unabdingbar.

Der ChainCUT-Quarantänerechner führt nach Erfassung der Kontakte eine automatische Risikoanalyse durch und erstellt konkrete individuelle Maßnahmenvorschläge. Damit steht den Verantwortlichen am Arbeitsplatz ein leicht zu bedienendes Tool zur Verfügung, das das für die Entscheidung und Einleitung optimaler Maßnahmen erforderliche medizinische und epidemiologische Fachwissen bereitstellt. In Kombination mit der Organisation einer betrieblich veranlassten Schnelltestung können alle zeitkritischen Maßnahmen zum frühestmöglichen Zeitpunkt eingeleitet werden. Damit ist dieses Tool expertengestützten zeitverzögerten Vorgehensweisen systematisch bei der schnellen Eingrenzung von Ausbrüchen überlegen.

## Fazit

Die neuen SARS-CoV-2-Virusvarianten können mit ihrer erhöhten Infektiosität auch am Arbeitsplatz große Ausbrüche verursachen, die die Beschäftigten und auch die Fortführung der Geschäftstätigkeit gefährden. Dabei verfügen Betriebe und Arbeitgeber über sehr effektive Interventionsmöglichkeiten wie die Organisation und verbindliche Anordnung von Schutzmaßnahmen sowie auch die Absonderung von Beschäftigten durch Verzicht auf die Arbeitsleistung vor Ort. Mit diesen Interventionsmöglichkeiten und dem vorgestellten erweiterten Risikomanagement ist es trotz der mit Tätigkeiten verbundenen Sozialkontakte möglich, die innerbetrieblichen Ansteckungsrisiken für Beschäftigte sehr gering zu halten.

Die gezielte risikobasierte Kombination von Basisschutz, betrieblichem Isolations- und Quarantänemanagement, Symptommonitoring und Teststrategie zeigt eine deutlich höhere Wirksamkeit als Einzelmaßnahmen. Diese Effizienz ist insbesondere bei kritischen Virusmutationen durch eine vorwärts gerichtete Absonderungsstrategie über mehrere Virusgenerationen und Frühinterventionen zu optimieren. Grundlage ist eine epidemiologische Modellierung mit differenzierter Risikoanalyse der Kontakte in Abhängigkeit von der tagesabhängigen Infektiosität. Damit können Risiken sehr viel besser eingeschätzt und daraus zielgenaue Maßnahmen abgeleitet werden.

Beispielrechnungen zeigen, dass der Zeitpunkt der Einleitung von Interventionen ein entscheidender Erfolgsfaktor ist. Da behördliche Kontaktverfolgungen in der Praxis häufig erst mit einem kritischen Zeitverzug beginnen können, stellt die sofortige arbeitsplatznahe Frühintervention eine entscheidende Verbesserung bei der schnellen Eingrenzung von Ausbrüchen dar. Der auf diesen Grundlagen entwickelte Quarantänerechner ermöglicht auch Laien eine differenzierte Risikoanalyse mit Einleitung optimierter Maßnahmen. Gezielte Teststrategien und Alternativmaßnahmen stellen die Personalverfügbarkeit sicher.
